# Density functional theory-based study on the structural, electronic and spectral properties of gas-phase PbMg_*n* ^−^_ (*n* = 2–12) clusters

**DOI:** 10.1098/rsos.240814

**Published:** 2024-09-18

**Authors:** Zai-Fu Jiang, Ding-Mei Zhang, Pan-Long Kong, Jing-Jing Wang, Wei Dai, Ben-Chao Zhu

**Affiliations:** ^1^ School of Mathematics and Physics, Jingchu University of Technology, Jinmen 448000, People’s Republic of China; ^2^ School of Public Health, Hubei University of Medicine, Shiyan 442000, People’s Republic of China

**Keywords:** DFT, gas-phase PbMg_n^−^_ clusters, CALYPSO, structural and spectral properties

## Abstract

Gas-phase PbMg_*n^−^*_ (*n* = 2–12) cluster structures were globally searched on their potential energy surfaces by means of the CALYPSO prediction software. Structural optimization and calculations of properties such as relative energy and electronic structure were then carried out by density functional theory for each size of low energy isomer. The structural, relative stability, natural charge population, natural electronic configuration and distribution of the strongest peaks of the infrared and Raman spectra of the low energy isomers of PbMg_*n^−^*_ (*n* = 2–12) clusters were systematically investigated in the present work. It was shown that the PbMg_7^−^_ cluster ground state isomer exhibits the highest stability, for which special electronic excitation and chemical bonding analyses were performed. It is reasonable to believe that this work enriches the structural, spectroscopic and other data of magnesium-based clusters and provides some theoretical basis for possible future experimental syntheses.

## 1. Introduction

The study of sub-nanometre-sized clusters of a few to several tens of Mg and Mg-based doped atoms has attracted a great deal of interest from researchers in recent years [1–10]. This is because, on the one hand, atomic clusters at this size have structural diversity and, on the other hand, they are in an intermediate physical state between atoms and nanoparticles, and their physical and chemical properties present interesting size dependencies that are necessary for one to understand the transition of matter from individual atoms to bulk macroscopic matter. In addition, inspired by the study of gold cluster assembled nanomaterials [11,12], assembled Mg-based nanoparticles with specific Mg-based cluster structures as basic units are worthy of theoretical exploration. Such interest mainly stems from the versatility of sub-nanometre-sized particles, for example they tend to exhibit high activity and selectivity for catalytic applications [13,14], and, thus, many metal clusters can serve as potential base units for nanoelectronic or spintronic devices [15–18]. Mg-based clusters have extremely good properties in terms of hydrogen storage performance and are an important component of new hydrogen storage nanomaterials for the future [19–25]. In addition, atomic cluster studies of the semiconductor elements Si_*n*_ [26–28] and Ge_*n*_ [29–35] have greatly enriched the development of nanomaterials based on them. The key work in the study of small-sized atomic clusters is to obtain the structure of the lowest energy isomer for a determined number of atoms, which in turn can be researched by quantum mechanics-based calculations [36–39] to obtain its physical and chemical properties. In other words, the beginning of all studies on atomic clusters is based on their structures, and, therefore, various types of atomic clusters have been reported extensively.

Taking magnesium clusters as an example, because magnesium alloys have important applications in many fields, atomic clusters have provided many interesting results as potential nanomaterials of magnesium alloys. From the periodic table of elements, studies on various charge states of Be [40–44], Li [8], Na [9], Ni [45], Pd [46], Ge [47], Ga [48], Au [49] etc.-doped Mg_*n*_ (*n* is the number of magnesium atoms) clusters have presented results on the geometric growth, stability, charge transferability and theoretical spectral prediction of small-sized Mg-based clusters. These studies show that doping magnesium clusters with different numbers of atoms in arbitrary charged states is worth investigating. This is because, first, they can enrich, at least theoretically, the database of the Mg-based cluster family. Moreover, their structural size dependence and diversity provide corresponding support for the development of Mg-based nanomaterials. Many novel microstructures are theoretically predicted to exist in doped magnesium clusters, such as the cage-like structures of SiMg_8_ [50] and GeMg_8_ [47], the tower-like shape of BeMg_9_ [42], the sphere-like shape of BeMg_16_ [41], and the cage-like shape of PdMg_6_ [46]. In addition, chemical bonding analysis of these structures shows that metal bonds are not present in the small-sized magnesium clusters, which instead are dominated by ionic bonds. The present work, on anionic Pb-doped Mg_*n*_ (*n* = 2–12) clusters, is to some extent a continuation of the work on Mg-based clusters. PbMg_*n^−^*_ (*n* = 2–12) clusters are systematically studied, including the growth mechanisms of cluster size, relative stability, charge transfer, chemical bonding and spectroscopic properties of the lowest energy isomers of each size cluster on its potential energy surface. This research not only adds new members to the family of Mg-based clusters but also provides theoretical data to guide future experimental validation.

## 2. Methods

Since the atomic clusters are based on the physical background of atomic sub-nanometre-sized atomic aggregation, they must be calculated by quantum mechanical methods. The initial structures of the PdMg_*n^−^*_ (*n* = 2–12) clusters were searched on the potential energy surface using the CALYPSO quantum chemistry prediction software [51]. CALYPSO is a powerful crystal structure prediction program that has been successfully applied for nearly a decade to predict the structure of clusters [52–54], two-dimensional layers [55] and three-dimensional crystals [56]. CALYPSO is based on a particle swarm optimization (PSO) algorithm that performs global searching on potential energy surfaces for a given chemical composition and external conditions such as pressure to obtain low energy isomers’ structures. Specifically, an extensive global minimum search was performed using the CALYPSO code for anionic PbMg_*n*_ clusters in the range 2 ≤ *n* ≤ 12. First, 50 initial structures were randomly generated under symmetry conditions, and in each subsequent generation of optimization, 80% of the new structures were generated based on a PSO algorithm that selects the previous generation of structures with high fitness, while the remaining 20% were randomly generated. As the number of generations increases, the exclusion of similar structures is achieved through characterization matrices. Overall, isomers of PbMg_*n^−^*_ were generated with 20 structures per generation for a total of 50 generations; therefore, 1000 structures were obtained at each size at B3LYP functional [57] and low-level 6-31G basis set [58] using the Gaussian 09 code [59]. Second, a higher level of structural optimization was performed for the 50 lowest energy isomers of the 1000 structures generated by CALYPSO, with the 6-311G(d) basis set [58] for the Mg atom and the lanl2dz pseudopotential basis set [60] for the Pb atom. The choice of this calculation level is based on successful reports of Mg-based clusters in recent years [44,47,50,61–63]. Based on the total number of electrons for each size of PbMg_*n^−^*_, four spin multiplicities of 2, 4, 6 and 8 were considered for the optimization of each isomer. In addition, the structural optimization was accompanied by vibration frequency calculations to ensure that the resulting structure is a local minimum energy state on its potential energy surface. The charge transfer properties, such as natural charge population (NCP) and natural electron configuration (NEC), were obtained by a natural bond orbital (NBO) method [64]. The electronic excited state analysis of the most stable cluster was studied by time-dependent density functional theory (DFT) calculations of the 50 excited states [65]. The chemical bonding analysis was performed by electron localization function (ELF) [66] calculation of the ELF values for the Pb–Mg and Mg–Mg topological critical points. Multiwfn software [67,68] was used in this research work for ELF, UV-Vis, hole–electron analysis and distribution maps of electronically excited states, as well as for density of states (DOS) for PbMg_7^−^_.

## 3. Results and discussion

### 3.1. The structural growth of PbMg_*n^−^*_ (*n* = 2–12) clusters

**Figure 1.**
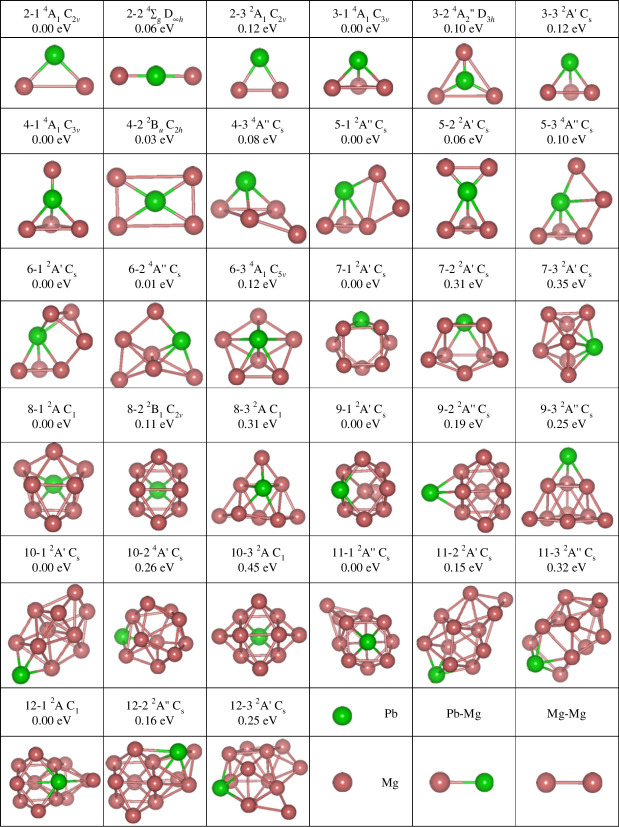
Isomer *n*-*i* structures for the three lowest energy clusters; *n* is the size of PbMg_n^−^_ (*n* = 2–12) clusters, *i* (*i* = 1–3) is the energy minimum label for each size cluster. The energies presented are the energy differences (eV) of the ground state isomer of their corresponding sizes.

Figure 1 shows a total of 33 isomers named *n-i*, where *n* is the number of Mg atoms and *i* = 1, 2, 3, corresponding to the ground, second and third lowest energy states, respectively. As shown in table 1, the lowest vibrational frequencies of the isomers are positive, indicating that they meet the requirement that there can be no imaginary frequencies in the frequency calculations, i.e. all optimized isomers are not excited states but local lowest energy states on the potential energy surface. In addition, each isomer of the PbMg_*n^−^*_ (*n* = 2–12) cluster is shown with its point group symmetry, electronic states and their energy differences from the ground state. In table S1 of the Supplementary Material, we also show the atomic coordinates of the radical isomers of each cluster for the reader’s use. The three lowest energy isomers of the PbMg_2^−^_ cluster are the isosceles triangular shapes 2-1 (C_2*v*_, ^4^A_1_) and 2-3 (C_2*v*_, ^2^A_1_), and the linear shape 2-2 (D_∞*h*_, ^4^Σ_g_). The ground state isomer has 0.06 and 0.12 eV lower energy than the isomers 2-2 and 2-3, respectively. The ground state isomer 3-1 (C_3*v*_, ^4^A_1_) and the third lowest energy isomer 3-3 (C_S_, ^2^A′) of the PbMg_3^−^_ cluster possess a tetrahedral structure, while 3-2 (D_3*h*_, ^4^A_2_″) exhibits a planar geometry with its Pb atom located at the centre of the triangle. In addition, the isomers 3-3 and 3-2 have 0.12 and 0.10 eV higher energy than their ground state isomers, respectively. For the PbMg_4^−^_ cluster, the second lowest energy isomer 4-2 (C_2*h*_, ^2^B_*u*_) still maintains a planar structure, where the Pb atom is located at the centre of a rectangular structure formed by four Mg atoms. The isomers 4-1 (C_3*v*_, ^4^A_1_) and 4-3 (C_S_, ^4^A″), on the other hand, are based on the growth of the tetrahedral structure 3-1, with one Mg atom adsorbed in different directions. The energy of the ground state isomer 4-1 is 0.03 and 0.08 eV lower than that of the isomers 4-2 and 4-3. For the PbMg_5^−^_ cluster, calculations reveal that isomers 5-1 (C_S_, ^2^A″), 5-2 (C_S_, ^2^A′) and 5-3 (C_S_, ^4^A″) are obtained by adsorption of two Mg atoms in different orientations by isomer 3-1 or based on adsorption of one Mg atom in different orientations by isomer 4-1 while slightly changing the original structure. A similar situation occurs in the formation of the three lowest energy isomeric structures of PbMg_6^−^_. Based on the tetrahedral structure of isomer 3-1 adsorbing three Mg atoms at different positions or fine-tuning its own structure based on the structure of isomer 5-1 while adsorbing one Mg atom, the geometrical structures of isomers 6-1 (C_S_, ^2^A′), 6-2 (C_S_, ^4^A″) and 6-3 (C_5*v*_, ^4^A_1_) can be searched. In addition, the second lowest and third lowest energy isomers of PbMg_5^−^_ and PbMg_6^−^_ can be found at energies above their ground state isomers of 0.16, 0.10, 0.01 and 0.12 eV, respectively. Starting from the PbMg_7^−^_ cluster, a new structural growth pattern emerges. The isomer 7-1 (C_S_, ^2^A′) is a staggered stack of two planar rectangles, while the isomers 7-2 (C_S_, ^2^A′) and 7-3 (C_S_, ^2^A′) are structures formed by the adsorption of three Mg atoms based on a pentahedral structure formed by five atoms. The energies of isomers 7-2 and 7-3 are 0.31 and 0.35 eV higher than their ground states, respectively. The basic structures of isomers 8-1 (C_1_, ^2^A), 8-2 (C_1_, ^2^A), 9-1 (*C*_S_, ^2^A′), 9-2 (C_S_, ^2^A″), 10-1 (C_S_, ^2^A′), 10-3 (C_1_, ^2^A), 11-1 (C_S_, ^2^A″), 11-3 (C_S_, ^2^A″), 12-1 (C_1_, ^2^A) and 12-2 (C_S_, ^2^A″) are almost identical and can be seen as adsorption of another atom at the apex of the structure of isomer 7-1. This basic unit structure is presented in electronic supplementary material, figure S1, but it is worth noting that the position of the Pb atom is often not fixed in the clusters. The structures of isomers 8-3 (C_1_, ^2^A), 9-3 (C_S_, ^2^A″), 10-2 (C_S_, ^4^A′), 11-2 (C_S_, ^2^A′) and 12-3 (C_S_, ^2^A′), on the other hand, show an irregular cage-like geometry. Furthermore, the calculations show that the second lowest energy and third lowest energy isomers of the PbMg_*n^−^*_ (*n* = 8–12) cluster have energies 0.11, 0.31, 0.19, 0.25, 0.26, 0.45, 0.15, 0.32, 0.16 and 0.25 eV higher than their ground state counterparts.

**Table 1.** The bonding energy per atom: *E*_b_, second-order difference energy; ∆_2_ *E*, HOMO–LUMO energy gap (*E*_gap_); the lowest vibrational frequency; Pb atomic natural charge population (NCP-Pb) and natural electron configuration (NEC-Pb) in the lowest energy state of PbMg_*n^−^*_ (*n* = 2–12) clusters.

cluster	*E*_b_ (eV)	∆_2_ *E* (eV)	α-*E*_gap_ (eV)	β-*E*_gap_ (eV)	lowest vib. freq. (cm^−1^)	NCP-Pb (e)	NEC-Pb (e)
PbMg_2^−^_	0.48	–	2.03	2.98	23	−0.90	[core]6s^1.91^6p^2.98^
PbMg_3^−^_	0.49	0.17	1.99	2.86	34	−1.18	[core]6s^1.86^6p^3.32^
PbMg_4^−^_	0.46	−0.11	1.90	2.47	12	−1.53	[core]6s^1.85^6p^3.67^7p^0.01^
PbMg_5^−^_	0.45	0.06	1.13	1.40	9	−1.12	[core]6s^1.77^6p^3.35^
PbMg_6^−^_	0.44	−0.67	1.07	1.20	15	−1.23	[core]6s^1.74^6p^3.49^
PbMg_7^−^_	0.52	0.43	1.66	1.29	15	−0.87	[core]6s^1.71^6p^3.16^
PbMg_8^−^_	0.53	0.31	1.27	1.86	28	−0.79	[core]6s^1.68^6p^3.10^
PbMg_9^−^_	0.51	0.00	1.19	1.88	23	−0.83	[core]6s^1.68^6p^3.15^
PbMg_10^−^_	0.49	−0.31	1.25	1.11	41	−0.74	[core]6s^1.70^6p^3.04^
PbMg_11^−^_	0.50	0.40	1.28	1.12	57	−0.76	[core]6s^1.68^6p^3.08^
PbMg_12^−^_	0.48	–	1.09	1.04	35	−0.79	[core]6s^1.68^6p^3.10^

In fact, the growth of small-sized Mg-based clusters based on tetrahedral unit and tower-like unit structures has been reported in the study of Be-, Ga-, Ge-, Si-, Na- and other atomic or ion-doped magnesium clusters. This suggests that the geometric structure of small- and medium-sized PbMg_*n^−^*_ (*n* = 2–12) clusters does not vary much with respect to these alkali metals.

### 3.2. The stability properties

As a ground state, the lowest energy isomers of PbMg_*n^−^*_ of different sizes in figure 1 are worthy of further investigation. The bonding energy per atom (*E*_b_), the second-order energy difference (∆_2_ *E*) and the highest occupied molecular orbital (HOMO)–lowest unoccupied molecular orbital (LUMO) energy gap (*E*_gap_) are calculated at the same level as B3LYP/6-311G(d) to study the relative stability of these ground state isomers. Formulas for these three energies, which measure the relative stability of clusters, are shown in equations (3.1)–(3.3) below:


(3.1) $$E_{b}\left({\text{PbMg}}_{n}^{-}\right)=\frac{nE\left(\text{Mg}\right)+E\left({\text{Pb}}^{-}\right)-E\left({\text{PbMg}}_{n}^{-}\right)}{n+1},$$

(3.2) $$\Delta_{2}E\left({\text{PbMg}}_{n}^{-}\right)=E\left({\text{PbMg}}_{n+1}^{-}\right)+E\left({\text{PbMg}}_{n-1}^{-}\right)-2E\left({\text{PbMg}}_{n}^{-}\right),$$

(3.3) $$E_{\text{gap}}\left({\text{PbMg}}_{n}^{-}\right)=E_{\text{LUMO}}\left({\text{PbMg}}_{n}^{-}\right)-E_{\text{HOMO}}\left({\text{PbMg}}_{n}^{-}\right),$$

where *E* (eV) represents the energy of the object in the parentheses on its right side. The energy difference between the HOMO and the LUMO is *E*_gap_.

**Figure 2.**
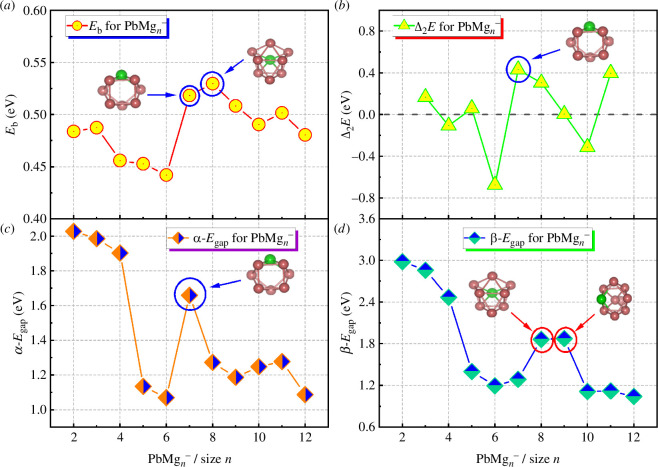
Relative stability energies, (*a*) *E*_b_, (*b*) Δ_2_ *E*, (*c*) *E*_gap_ for α electrons (α-*E*_gap_) and (*d*) *E*_gap_ for β electrons (β-*E*_gap_) in the lowest energy state isomers of PbMg_*n^−^*_ (*n* = 2–12) clusters.

Table 1 shows the results for these energies, and their size-dependent curves are plotted in figure 2. As shown in table 1 and figure 2*a*, the *E*_b_ values of each size isomer are in the range 0.44–0.53 eV. The *E* **_b_** curves show oscillations with increasing cluster size, with PbMg_6^−^_ (0.44 eV) having the smallest *E*_b_ value and PbMg_8^−^_ (0.53 eV) the largest, followed by PbMg_7^−^_ (0.52 eV). This implies that PbMg_7^−^_ and PbMg_8^−^_ are more stable relative to other clusters. According to equation (3.2), the Δ_2_ *E* value of a cluster is an important parameter to measure its relative stability compared with its neighbour, and the Δ_2_ *E* curve in figure 2*b* shows that PbMg_6^−^_ (−0.67 eV) and PbMg_7^−^_ (0.43 eV) have the smallest and the largest values, respectively, indicating that PbMg_7^−^_ possesses the highest local stability. *E*_gap_ in equation (3.3) can be used to measure the chemical stability. Since the PbMg_*n^−^*_ cluster is an open-shell system, it does not have the same number of α and β electrons, as shown in table 1, as well as figure 2*c*,*d*, both α-*E*_gap_ and β-*E*_gap_ show oscillatory behaviours, with a local maximum of the α-*E*_gap_ value for PbMg_7^−^_ (1.66 eV) and local maxima of β-*E*_gap_ occurring at PbMg_8^−^_ (1.86 eV) and PbMg_9^−^_ (1.88 eV), which implies that their chemical stability is relatively high. Overall, the above calculations indicate a relatively high stability of the ground state isomer of the PbMg_7^−^_ cluster. Since this higher stability isomer will be studied in more depth later, its side view and atomic numbering are shown in electronic supplementary material, figure S1.

**Figure 3.**
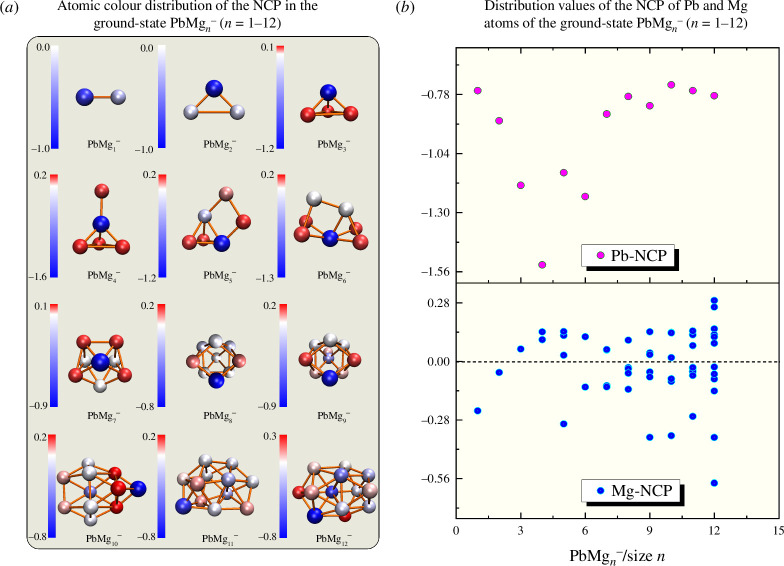
Natural charge population (NCP) in the lowest energy state PbMg_*n^−^*_ (*n* = 1–12): (*a*) atomic colour distribution figures and (*b*) atomic distribution values.

### 3.3. Natural charge population and natural electron configuration analysis

**Figure 4.**
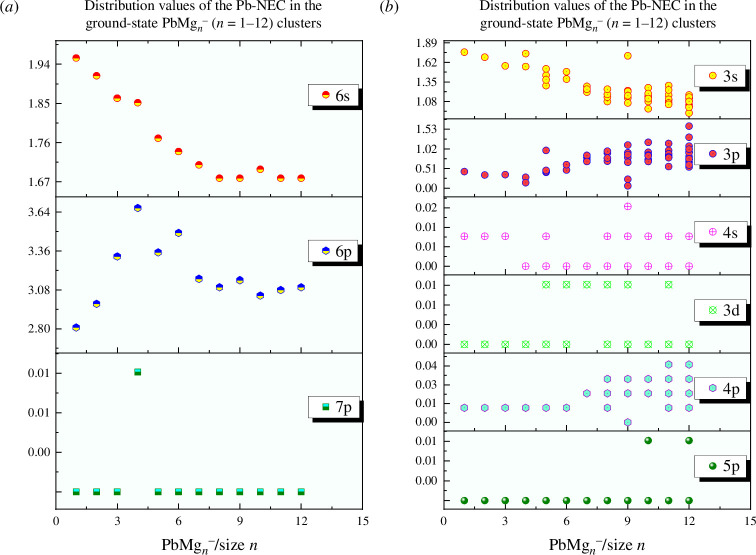
Natural electron configuration (NEC) in the lowest energy state PbMg_*n^−^*_ (*n* = 1–12): (*a*) Pb distribution values in 6s, 6p and 7p shells, and (*b*) Mg distribution values in 3s, 3p, 4s, 3d, 4p and 5p shells.

NBO calculations on all the ground state isomers of PbMg_*n^−^*_ yielded their NCP and NEC, both of which are useful for gaining insights into the properties of the electronic structure of clusters. In order to better discuss the charge transfer and electronic configuration properties, the PbMg_1^−^_ cluster ground state isomer is added to the study in this section. The NCP and NEC calculations for Pb atoms are shown in table 1, while those for all Mg atoms are shown in electronic supplementary material, tables S2 and S3, and figures 3 and 4 graphically plot them. Figure 3*a* shows the colour distribution of NCP values for Pb and Mg atoms, where blue indicates the gain of electrons while red is the loss of electrons. Obviously, it shows that all 12 Pb atoms in PbMg_*n^−^*_ (*n* = 1 – 12) clusters always gain electrons in the range of −1.53*e* to −0.74*e* (*e* stands for electrons). Figure 3*a,b* indicates that 41 out of 78 Mg atoms in PbMg_*n^−^*_ (*n* = 1–12) clusters gain electrons in the range 0.02e to 0.29e, and the remaining 37 Mg atoms lose electrons in the range −0.58*e* to −0.03*e*. The electron transfer properties of Mg atoms are non-uniform because the object of our study is negatively charged. In similar studies reported for other, neutral Mg-based clusters, the Mg atom always plays the role of losing electrons. Because the electronegativity of the Mg atom (1.31) is less than that of the Pb atom (2.33), it seems reasonable for Pb to get an electron, but since PbMg_*n^−^*_ as a whole is negatively charged, it is normal for some of the Mg atoms to get electrons as well.

As shown in figure 4*a,b*, the NEC distributions of the 6s, 6p and 7p valence layers of the Pb atom and the 3s, 3p, 4s, 3d, 4p and 5p valence layers of Mg atoms exhibit certain regularities. First, compared with the NECs of [core]6s^2^6p^2^ and [core]3s^2^ for bare Pb and Mg atoms, respectively, calculations show that the Pb of the ground state isomers of PbMg_*n^−^*_ clusters always loses electrons in the 6s valence layer and gains electrons in the 6p valence layer, with the exception of PbMg_4^−^_, whose Pb atom gains 0.01*e* electrons in the 7p shell layer. The Mg atoms always lose electrons in the 3s valence layer, gain most electrons in the 3p valence layer and gain very few electrons in the 4s, 3d, 4p and 5p shell layers. An important conclusion is that the 6p shell layer NEC distribution of Pb atoms confirms the formation of PbMg_*n^−^*_ (*n* = 1–12) clusters based on anionic Pb-doped Mg_*n*_ clusters. Second, during PbMg_*n^−^*_ cluster formation, both Pb and Mg atoms undergo hybridization of sp orbitals, with Mg atoms being more deeply hybridized.

### 3.4. Infrared and Raman spectra

**Figure 5.**
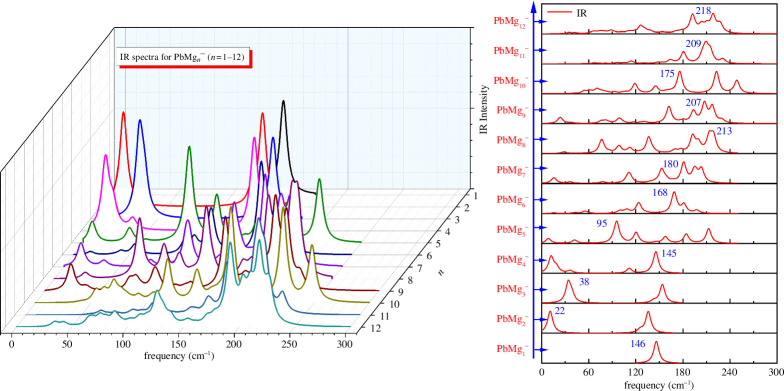
Infrared (IR) spectra for the lowest energy state PbMg_*n^−^*_ (*n* = 1–12) clusters and the strongest peaks distributions.

**Figure 6.**
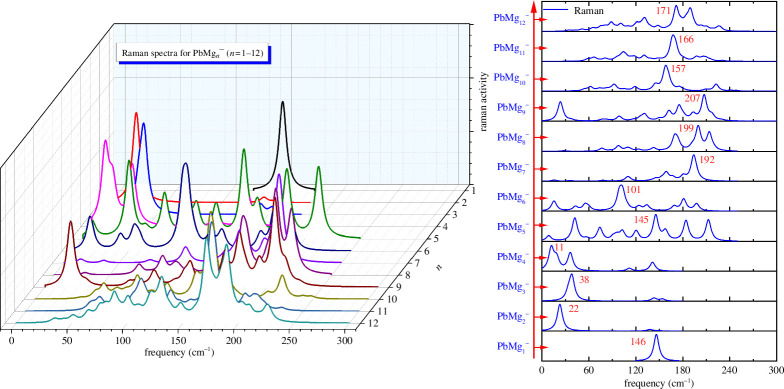
Raman spectra for the lowest energy state PbMg_*n^−^*_ (*n* = 1–12) clusters and the strongest peaks distributions.

The structure of the clusters determines their spectra, so spectral predictions can provide data to guide possible future experiments. As shown in figures 5 and 6, we have theoretically predicted and plotted the infrared (IR) and Raman spectra of the ground state isomers of all PbMg_*n^−^*_ (*n* = 1–12) clusters. Overall, the IR and Raman spectral peaks of PbMg_*n^−^*_ (*n* = 1–12) clusters are distributed in the 10–220 cm^−1^ frequency band. It is found that for PbMg_1^−^_ the only IR and Raman peaks are at 146 cm^−1^. PbMg_2^−^_ has two IR peaks and one Raman peak, with both the strongest IR peak and the Raman peak occurring at the very low frequency of 22 cm^−1^. The strongest IR and Raman peaks of PbMg_3^−^_ both appear at 38 cm^−1^. PbMg_4^−^_ possesses two distinct IR peaks and three Raman peaks, with the strongest IR and Raman peaks located at 145 and 11 cm^−1^, respectively. Calculations show that PbMg_5^−^_ has at least six detectable IR and Raman peaks distributed between 10 and 200 cm^−1^, with the strongest IR and Raman peaks occurring at 95and 145 cm^−1^, respectively. The strongest IR and Raman peaks of PbMg_6^−^_ can be found at 168 and 101 cm^−1^. The strongest IR and Raman peaks of PbMg_7^−^_ are located at 180 and 192 cm^−1^, respectively. The strongest IR and Raman peaks of PbMg_8^−^_ can be detected at 213 and 199 cm^−1^. The strongest IR and Raman peaks of PbMg_9^−^_ are both neatly located at 207 cm^−1^.The strongest IR and Raman peaks of PbMg_10^−^_ are detected at 175 and157 cm^−1^.The strongest IR and Raman peaks of PbMg_11^−^_ are located at 209 and 166 cm^−1^. Finally, the strongest IR and Raman peaks of PbMg_12^−^_ are found at 218 and 171 cm^−1^.

In conclusion, as shown in figures 5 and 6, although the theoretically calculated values of the strongest IR and Raman peaks of each cluster are very clear, the IR or Raman spectra exhibit a multi-peak nature as the size of the clusters increases, which can cause some difficulties in detecting cluster size directly through spectroscopic experiments. However, the theoretical data of these spectra can provide reference for the corresponding experimental spectra.

### 3.5. Further studies on the PbMg_7^−^_ cluster ground state isomer

#### 3.5.1. Ultraviolet–visible spectrum and excited state analysis

**Figure 7.**
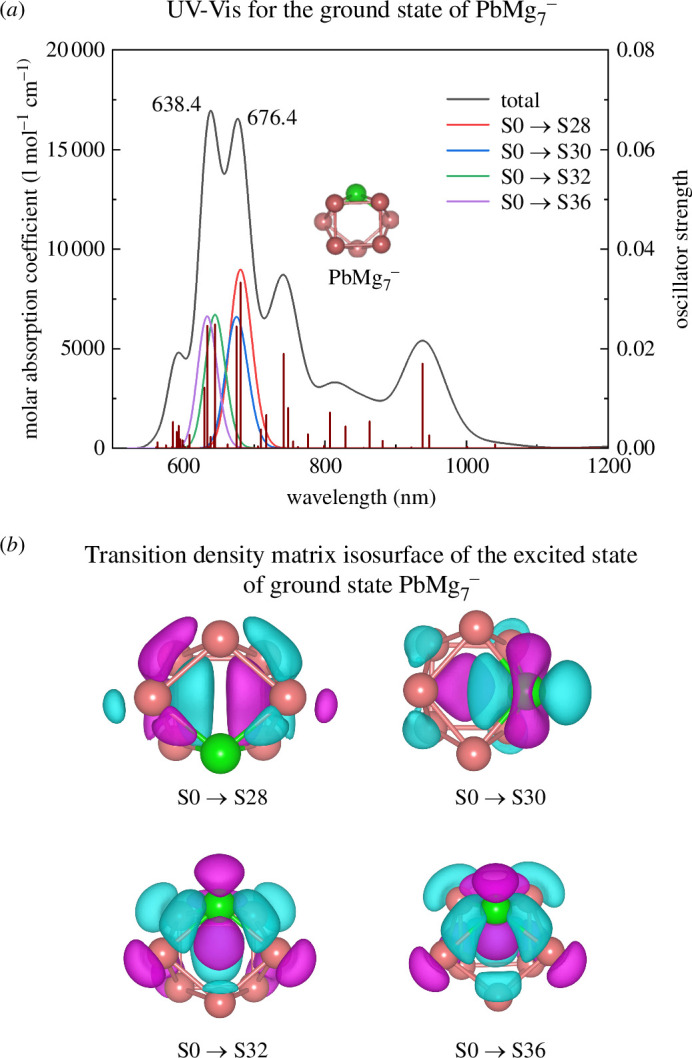
Excited state properties for the lowest energy state isomer of PbMg_7^−^_: (*a*) UV-Vis spectrum and (*b*) transition density matrix (TDM) isosurfaces for the S28, S30, S32 and S36 excited states.

PbMg_7^−^_ is worthy of further investigation as a cluster with relative overall excellent stability. Although its IR and Raman spectra have been calculated in the previous section, this section continues with a special look at its ultraviolet–visible (UV-Vis) spectrum. UV-Vis spectroscopy is another useful experimental tool for understanding the structure of matter and is therefore always used in the study of clusters. Figure 7*a* shows the UV-Vis absorption spectral curve of the ground state isomer of PbMg_7^−^_, and the vertical short straight lines within the curve indicate the oscillator strength (right-side *y*-axis). There are two strong peaks with close intensity at 638 and 676 nm, whose oscillator strength reaches 0.025 and 0.035, respectively. Excited state analysis shows that the main contributors to these two strong peaks are from the S0 → S28, S0 → S30, S0 → S32 and S0 → S36 transitions. In order to further investigate the properties of these four excited states, a hole–electron-based study of the excited states was carried out. Specifically, electronic supplementary material, table S4 shows the analysis of holes and electrons for the four most dominant excited states in the strongest peaks of the UV-Vis spectrum, including the distance between the centre of mass of the holes and electrons (*D*), the overall average distribution of electrons and holes (*H*), the overlap between the electron–hole distributions (*S*_r_), the degree of separation between the holes and the electrons (*t*), the hole-domain departure indices (HDI) and the electron-domain departure indices (EDI). The transition density matrix (TDM) distribution of the excited state is plotted in figure 7*b*, while electronic supplementary material, figure S2 shows the hole–electron distribution of the excited state, the hole–electron overlap function and the heat map of the overlap function and TDM distribution. As shown in electronic supplementary material, table S4, the *t*-parameter is less than zero and the *S*_r_-parameter is close to 1.0, indicating that there is no significant separation of holes and electrons during the electronic excitation of the four excited states to S28, S30, S32 and S36, while the *D*-parameter, HDI and EDI indices are relatively small, suggesting that the dominant peaks of the UV-Vis absorption spectrum of the PbMg_7^−^_ cluster ground state isomer are globally excited (GE). In addition, the TDM distribution in figure 7*b* and the hole–electron distribution and *S*_r_ distribution plots in electronic supplementary material, figure S2*a*,*b* graphically verify the above electronic excitation properties. To further demonstrate the contribution of each atom to the electronic excitation, electronic supplementary material, figure S2*c*,*d* shows the hole–electron distribution as well as the TDM by means of heat maps. An obvious conclusion is that the Pb atom contributes much more to electronic excitation than do holes, and the asymmetry of the diagonal part of the thermogram in the TDM heat map confirms that the cluster is GE.

#### 3.5.2. Chemical bonding analysis

**Figure 8.**
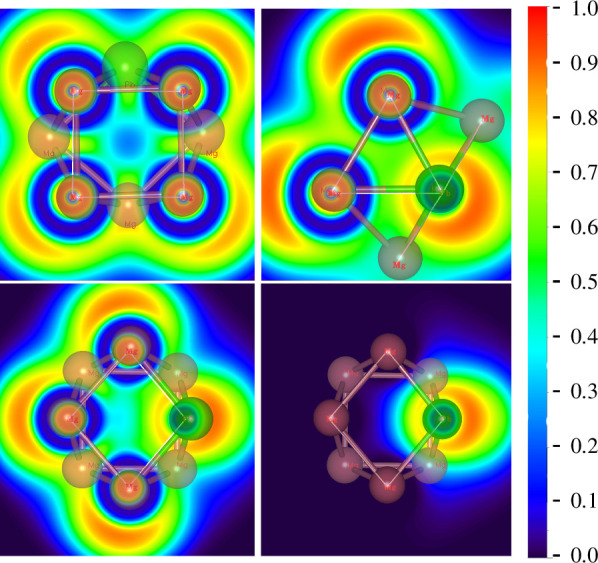
ELF analysis for the lowest energy state isomer of PbMg_7^−^_.

Since the nature of the chemical bonding of metal clusters may vary with size, we performed bonding analyses by ELF for the PbMg_7_^−^ cluster ground state isomer. The ELF values for Pb–Mg and Mg–Mg bonding sites were calculated and are plotted graphically in electronic supplementary material, table S5 and figure 8. Calculations show that all four Pb–Mg ELF values are <0.5, while all eight Mg–Mg ELF values are >0.5, indicating that the Pb–Mg bond in the PbMg_7^−^_ cluster ground state isomer is noncovalent, while the Mg–Mg bond is covalent. Combined with the NCP calculations in table 1 and electronic supplementary material, table S2, we can further conclude that Pb1–Mg4, Pb1–Mg5, Pb1–Mg6 and Pb1–Mg8 are ionic bonds. This result agrees with previously reported chemical bonding analyses of Mg-based clusters, such as PdMg_*n*_ [46], AuMg_*n*_ [49] and BeMg_*n*_ [47,62]. These findings confirm that even when metal atoms are doped into small-sized magnesium clusters, their chemical bonding has no metal bonding properties.

#### 3.5.3. Total density of states and partial density of states

**Figure 9.**
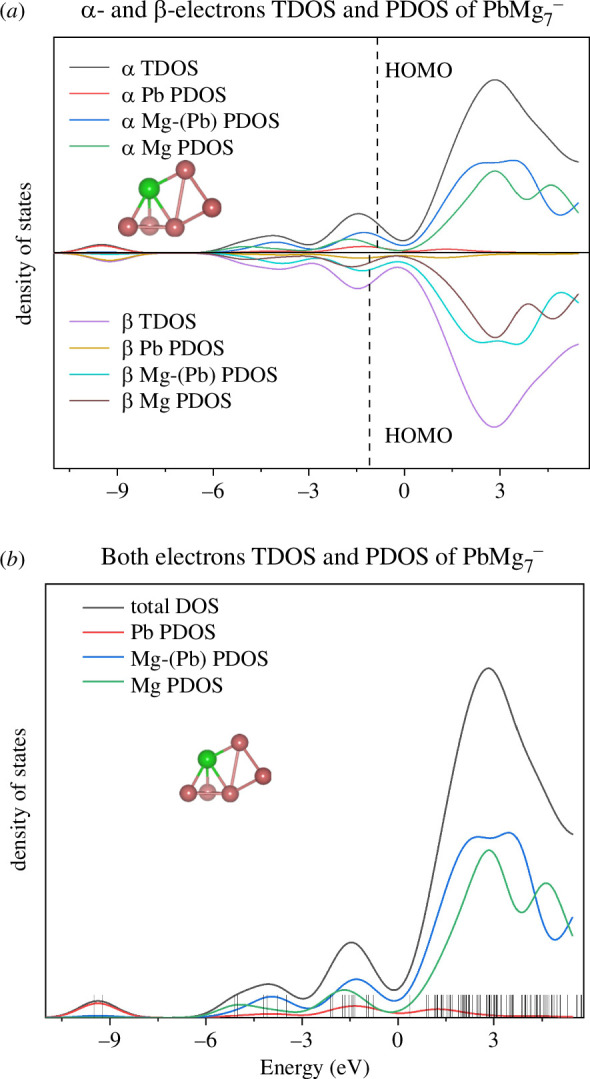
Total density of states (TDOS) and partial density of states (PDOS) analysis for the lowest energy state isomer of PbMg_7^−^_.

Atomic clusters, as isolated systems, have discrete energy levels, so in principle, DOS analysis is not possible, but if the discrete energy levels are artificially broadened into curves, DOS diagrams can be a useful tool for analysing the nature of the electronic structure of clusters. Here, we have performed total density of states (TDOS) and partial density of states (PDOS) calculations for the ground state isomer of the PbMg_7^−^_ cluster and plotted them graphically by means of Multiwfn, as shown in figure 9. In figure 9*a*, we show the TDOS and PDOS of the α and β electrons of PbMg_7^−^_, and the PDOS is defined by the different atomic contributions, where Mg-(Pb) represents the Mg atoms that are bonded to the Pb atom, and the rest of the Mg atoms are not bonded to Pb. Figure 9*b*, on the other hand, combines the contributions of the two electrons, along with the corresponding molecular orbitals (MOs; each shown by a discrete vertical line) energy levels on display. It was shown that Pb atoms contribute most to the two MOs with the lowest energies, the MOs with slightly higher energies are mainly contributed to by the three Mg atoms that are not bonded to Pb atoms, followed by the four Mg atoms bonded to Pb, and the main contributors to the MOs with higher energies, close to the HOMOs, are the Mg-(Pb) and to a lesser extent Mg, a property that holds for all the unoccupied molecular obitals (UMOs). Combining the discussions in §3.3 about NCP and NEC, we can clearly see that the lowest energy MOs of the PbMg_7^−^_ cluster are occupied by the 6p valence electrons of the Pb atoms and the 3s valence electrons of Mg-(Pb), and the electron occupancy of the MOs with higher energies is populated by the corresponding valence electrons of Mg-(Pb) and those of Mg. In other words, the strong p–d hybridization is responsible for the stability of PbMg_7^−^_. TDOS and PDOS analyses of the other clusters PbMg_*n^−^*_ (*n* = 2–6, 8–12) lead to similar conclusions as for the PbMg_7^−^_ isomers, as described in electronic supplementary material, figure S3.

## 4. Conclusion

Using the CALYPSO cluster structure search software, this work presents a systematic study of gas-phase anionic Pb-doped Mg_*n*_ (*n* = 2–12) clusters. The geometrical structural features of the three lowest energy isomers of different-size clusters are investigated, and the relative stability of the size dependence is calculated through several characteristic energies. It is shown that the _PbMg7^−^_ cluster ground state isomer has the highest integrated stability, and its excited state properties and chemical bonding properties are investigated in §3.5. In addition, the electronic structure of the cluster ground state isomers of various sizes is investigated through NCP and natural electronic configuration, while theoretical calculations predict where the strongest peaks of their IR and Raman spectra would appear.

## Data Availability

The Cartesian coordinates and energies of the most stable structures of all PbMg_n^−^_ (*n* = 2–12) clusters are provided in the online electronic supplementary material [69].
